# Development of a risk-stratified follow-up strategy based on early and late recurrence risk factors in locally advanced esophageal squamous cell carcinoma after definitive chemoradiotherapy

**DOI:** 10.3389/fonc.2026.1903732

**Published:** 2026-09-23

**Authors:** Liang Hong, Jianjian Qiu, Yuling Ye, Yilin Yu, Zhiping Wang, Mingqiu Chen, Wei Zhang

**Affiliations:** 1 Department of Radiation Oncology, Clinical Oncology School of Fujian Medical University, Fujian Cancer Hospital, Fuzhou, China; 2 Department of Gynecology, Clinical Oncology School of Fujian Medical University, Fujian Cancer Hospital, Fuzhou, China; 3 Department of Radiotherapy, Cancer Center, The First Affiliated Hospital of Fujian Medical University, Fuzhou, Fujian, China

**Keywords:** ER and LR, follow-up strategy, machine learning and SHAP, risk factors, RMST

## Abstract

**Background:**

The high recurrence rate following definitive chemoradiotherapy (dCRT) for locally advanced esophageal squamous cell carcinoma (ESCC) remains a paramount clinical challenge. However, there is currently a lack of research to explore the different effects of inflammation-related indicators on early recurrence (ER) and late recurrence (LR).

**Methods:**

We retrospectively analyzed 905 locally advanced ESCC patients. Six machine learning algorithms were used for consensus feature selection, followed by multivariate Cox regression to identify independent prognostic factors for ER and LR. A prognostic nomogram was developed, and restricted mean survival time (RMST) quantified survival differences, guiding follow-up strategies.

**Results:**

Machine learning combined with Cox analysis identified RT dose, tumor thickness, and N stage as independent risk factors for ER, while concurrent chemotherapy, N stage, pan-immune-inflammation value (PIV), and platelet-to-albumin ratio (PAR) were identified for LR. Risk models effectively stratified patients into high-, moderate-, and low-risk groups with significantly distinct PFS and OS, and high-risk groups were enriched with adverse features including elevated N stage, PAR, PIV, and tumor burden. The developed nomogram demonstrated good calibration, discriminative ability (with 5-year OS and PFS AUC values of 0.738 and 0.771), and provided higher net clinical benefit than single variables. The C-index of the model was 0.652 and 0.677 for OS and PFS. Based on RMST analysis revealing a significant survival gradient across risk subgroups (5-year OS-RMST: 21.0 vs. 32.5 vs. 44.7 months for high-, moderate-, and low-risk groups, respectively), a risk-stratified follow-up strategy was developed, recommending intensive (every 1–3 months), step-down, and annual surveillance schedules for high-, moderate-, and low-risk patients.

**Conclusion:**

We developed and internally validated a prognostic nomogram based on inflammatory indicators that can effectively quantify risks and stratify them. Moreover, we formulated a novel risk-adaptive follow-up strategy framework based on RMST, providing potential decision-making tools for the individualized and precise management of ESCC. However, this strategy remains hypothesis-generating and requires external prospective validation before widespread clinical adoption.

## Introduction

Esophageal squamous cell carcinoma (ESCC) is a highly prevalent gastrointestinal malignancy with persistently high incidence and mortality rates worldwide, particularly in East Asia (1, 2). Due to its atypical early symptoms, over 60% of patients are already diagnosed at a locally advanced stage or with distant metastasis (3). For patients with unresectable locally advanced ESCC, definitive chemoradiotherapy (dCRT) serves as the standard non-surgical treatment, aiming to achieve disease cure and prolong survival (4, 5). Despite advances in multidisciplinary management in recent years, the overall survival (OS) outlook for these patients remains concerning (6). The current overall 5-year survival rate is low, and negative pathological tumor factors, treatment tolerance, and treatment-related adverse events significantly impact prognosis (7, 8). More critically, the high risk of recurrence and the diversity of recurrence patterns make the early identification of high-risk patients and the optimization of individualized follow-up and treatment strategies key to improving survival. Therefore, analyzing disease progression within different time windows after dCRT and its associated risk factors holds significant theoretical and clinical importance for improving survival outcomes in locally advanced ESCC.

Disease progression is not a monolithic event; different progression patterns profoundly influence patient prognosis and management strategies. For instance, Gao et al. categorized progression patterns in patients receiving chemoimmunotherapy into three types: enlargement of existing lesions only, appearance of solely new metastatic lesions, and both enlargement of existing lesions and appearance of new metastases, with corresponding patient survival progressively shortening (9). Similarly, Wu et al. classified progression patterns in patients receiving targeted therapy into slow progression, local progression, and rapid progression, with significantly different survival observed among the three groups (10). Furthermore, local recurrence versus distant recurrence, as well as different sites of recurrence, also significantly impact patient prognosis and clinical management (11, 12). Further distinguishing progression based on its timing is of critical clinical value. For example, using 24 months post-treatment as a cutoff to categorize progression into early (ER) and late recurrence (LR). ER typically indicates more aggressive tumor biology or primary treatment resistance and is associated with an extremely poor prognosis. In contrast, LR may be related to mechanisms such as tumor microenvironment evolution, immune escape, or reactivation of dormant tumor cells. Identifying the driving factors behind these two distinct progression modes is a prerequisite for achieving genuine individualized risk stratification, formulating intensified treatment plans, and optimizing surveillance strategies.

Currently, research specifically and systematically dedicated to discriminating between ER and LR and exploring their respective independent risk factors in patients with locally advanced ESCC receiving dCRT is lacking. To address this gap and to more comprehensively and robustly uncover the key factors influencing disease progression in different time windows post-dCRT, this study innovatively employs a two-stage analytical strategy. First, we integrate six machine learning algorithms based on different principles to perform preliminary screening and importance ranking of multi-dimensional variables encompassing clinicopathological characteristics, inflammation indices, and treatment parameters, aiming to obtain a consensus set of high-risk variables. Subsequently, these variables are incorporated into a multivariate Cox regression model for validation and confirmation. The objective is to establish independent risk factors significantly associated with ER and LR respectively, in patients with locally advanced ESCC undergoing dCRT. The findings of this study are expected to provide new evidence-based insights for constructing refined risk stratification models and achieving individualized clinical management.

## Materials and methods

### Patients

A total of 905 patients with locally advanced ESCC were enrolled in this study. All patients received dCRT or definitive radiotherapy (dRT) between January 2011 and December 2020. The inclusion criteria were as follows: (1) pathologically confirmed ESCC; (2) received dCRT or dRT; (3) tumor stage II-IVA according to the 8th edition of the UICC staging system; and (4) age ≥18 years. Patients were excluded if they met any of the following criteria: (1) incomplete clinical data; (2) history of other malignancies; or (3) loss to follow-up (Loss to follow-up was defined as the inability to contact the patient for more than six consecutive months after the expected follow-up timepoint, despite attempts via outpatient visits or telephone, resulting in missing information on survival or recurrence status). After screening, 905 patients were eligible and included in the final analysis. This study was conducted in accordance with the principles of the Declaration of Helsinki and was approved by the Ethics Committee of Fujian Cancer Hospital. Clinicopathological data were extracted from electronic medical records, including demographic characteristics (age, sex, height, weight), tumor features (location, thickness, length, T stage, N stage, TNM stage), treatment details (number of chemotherapy cycles, chemotherapy regimen, radiotherapy dose), and routine blood and biochemical parameters before treatment. Inflammatory and nutritional indices used in the study were calculated based on established formulas (detailed in **Supplementary Material 1**).

### Treatment

All patients received individualized radiotherapy, mainly using intensity-modulated radiotherapy (IMRT) or three-dimensional conformal radiotherapy (3D-CRT). The prescribed dose was 50–70 Gy, completed in 25–34 fractions. The target area delineation followed the NCCN guidelines: the gross tumor volume (GTV) included the primary tumor and metastatic lymph nodes; the clinical target volume (CTV) was placed 3 cm outward in the head-to-foot direction of the GTV and 0.5 cm outward in all other directions; the planning target volume (PTV) was 0.5–1 cm outward from the CTV (an additional 1 cm was added for the lymph node area). The plan required that 95% of the PTV volume reach the prescribed dose, and all dose-volume limits for organs at risk (OARs) were met. Patients received 0–9 cycles of chemotherapy based on platinum drugs, and the regimens were mainly divided into two groups: Group A was platinum-based regimens (such as paclitaxel/docetaxel combined with carboplatin, cisplatin, nedaplatin or lobaplatin); Group B was 5-FU-based regimens (5-FU + cisplatin). Blood routine, biochemical and electrocardiogram were monitored before and after each cycle, and the dose was adjusted in subsequent cycles when severe toxicity occurred.

### Study endpoints and follow-up

The primary endpoints of this study were progression-free survival (PFS) and overall survival (OS). PFS was defined as the time from the initiation of treatment to disease progression, death from any cause, or the last follow-up, whichever occurred first. OS was defined as the time from treatment initiation to death from any cause or the last follow-up.

All patients were followed according to a standardized schedule: every 3 months for the first 2 years after treatment, every 6 months for years 3 to 4, and annually thereafter from year 5 onward. Follow-up assessments included physical examination, laboratory tests (blood routine, biochemical, tumor markers), endoscopic examination (when necessary), and imaging studies (computed tomography of the neck, chest, and abdomen; brain magnetic resonance imaging; and positron emission tomography-computed tomography). In practice, the frequency of follow-up was based on the standard follow-up schedule recommended by clinical guidelines. The date of the final follow-up is January 2026.

### Definition of early and late recurrence

This study aims to analyze the risk factors associated with different recurrence patterns. Early and late recurrence were modeled as time-to-event outcomes rather than as baseline groups. In multiple solid tumors, 24 months is a commonly used and clinically meaningful threshold to differentiate early treatment failure from late recurrence (13–15). Furthermore, major guidelines such as NCCN and ESMO recommend intensive follow-up every 3 months during the first 2 years (24 months) after curative treatment, with extended intervals thereafter, indicating that the 24-month period represents the highest-risk window for recurrence. Therefore, with the 24-month mark after the start of radical treatment as the dividing point, only patients with documented recurrence were classified into these categories: patients with a recurrence time of 24 months or less were classified as early recurrence (ER, n = 411), and those with a recurrence time greater than 24 months were classified as late recurrence (LR, n = 145). Patients who never experienced recurrence throughout follow-up (n = 349) were treated as censored observations and were not assigned to either the ER or the LR group, because a censored observation is not a recurrence event and has no recurrence time to classify.

### Statistical analysis

All statistical analyses were performed using R software (version 4.5.2). A landmark analysis was first performed to justify the 24-month threshold for distinguishing early from late recurrence, by examining the distribution and cumulative proportion of recurrence events over time. Subsequently, receiver operating characteristic (ROC) curve analysis was employed to determine the optimal cut-off values for tumor length, tumor thickness, radiotherapy dose, number of chemotherapy cycles, body mass index (BMI), and inflammatory and nutritional indices. To identify high-risk factors associated with ER and LR, six machine learning algorithms were applied for feature selection: Random Forest, Extreme Gradient Boosting (XGBoost) (16), Gradient Boosting Machine (GBM) (17), ElasticNet (18), Support Vector Machine with Weighting (SVM_Weight) (19), and LASSO regression. Variables selected by three or more of these methods were then incorporated into cause-specific Cox proportional hazards models, in which early and late recurrence were modeled as separate competing events, to identify independent prognostic factors for each recurrence mode. To enhance the interpretability of the machine learning models, the SHapley Additive exPlanations (SHAP) method was used for visual explanation of the results.

A prognostic nomogram integrating the union of the ER and LR risk factors was constructed for predicting 1-, 3-, and 5-year OS and PFS. Discrimination was assessed by the concordance index (C-index) and time-dependent ROC analysis; calibration was evaluated by bootstrap-corrected calibration curves; and clinical utility was assessed by decision curve analysis (DCA). The incremental value of the full model over the ER and LR models was quantified by the category-free net reclassification index (NRI) and the integrated discrimination improvement (IDI). Survival differences were analyzed using Kaplan-Meier curves, and the restricted mean survival time (RMST) was calculated at 1 to 5 years (20). All tests were two-sided, and a p-value of less than 0.05 was considered statistically significant.

## Results

### Clinical characteristics

Among the 905 patients, 411 (45.4%) experienced early recurrence, 145 (16.0%) late recurrence, and 349 (38.6%) did not recur, of whom 127 (14.0%) died without documented recurrence and 222 (24.5%) remained alive and recurrence-free at the last follow-up. The cumulative incidence of early recurrence rose steeply to 45.3% at 24 months and then plateaued, whereas the cumulative incidence of late recurrence and of death without recurrence remained low throughout follow-up (**Figure 1A**). A landmark analysis further confirmed that the first 24 months constituted the highest-risk window for recurrence: 73.9% (411/556) of all recurrences occurred within 24 months after treatment, after which the recurrence hazard declined substantially and entered a plateau phase (**Figure 1B**).

**Figure 1:**
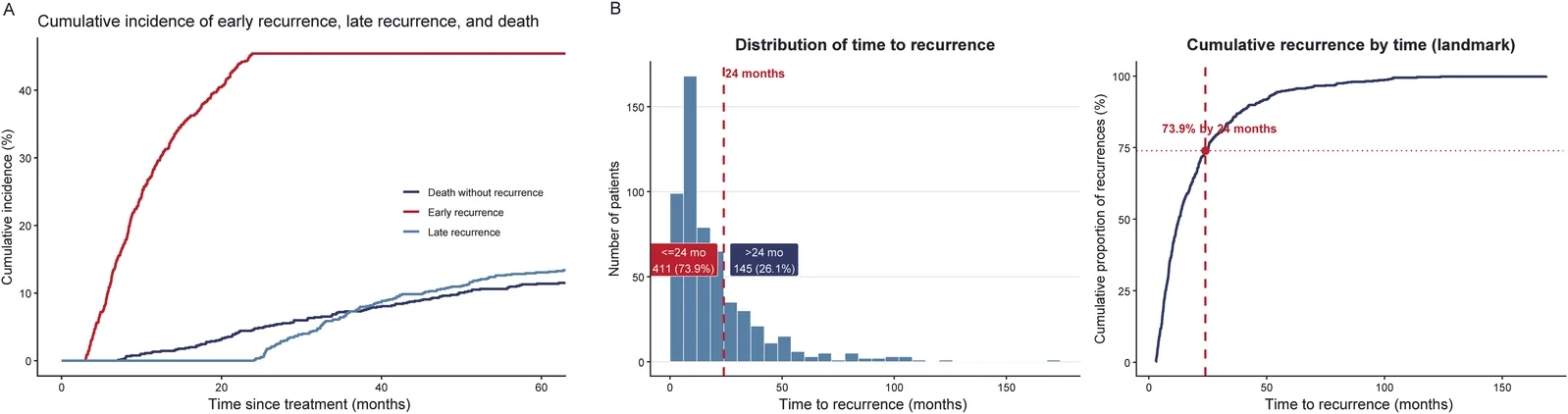
Recurrence patterns and justification of the 24-month cutoff. **(A)** Cumulative incidence curves of early recurrence (recurrence within 24 months), late recurrence (recurrence beyond 24 months), and death without recurrence estimated by the competing-risks method. **(B)** Landmark analysis supporting the 24-month threshold: distribution of time to recurrence (left) and cumulative proportion of recurrence events over time (right), showing that 73.9% (411/556) of all recurrences occurred within 24 months after treatment.

Baseline characteristics stratified by recurrence status are summarized in **Table 1**. Patients with early recurrence had a higher tumor burden, including greater tumor thickness (≥1.25 cm) and more advanced N stage (N2/3), than those with late recurrence or without recurrence. In addition, patients with early recurrence exhibited higher levels of systemic inflammatory and nutritional indices, including SII, SIRI, NLR, PLR, PIV, and PAR, whereas patients without recurrence had a higher BMI and a more favorable inflammatory-nutritional profile. These findings indicate that greater tumor burden and an unfavorable inflammatory-nutritional status at baseline are associated with a higher risk of early recurrence.

**Table 1:** Baseline characteristic analysis of the whole cohort, ER, LR, and no recurrence.

Variables		Total (N)	ER (411)	LR (145)	No recurrence (349)
Sex					
	Male	671 (74.1%)	322 (35.6%)	113 (12.5%)	236 (26.1%)
	Female	234 (25.9%)	89 (9.8%)	32 (3.5%)	113 (12.5%)
Age (years)					
	< 60	279 (30.8%)	144 (15.9%)	44 (4.9%)	91 (10.1%)
	≥ 60	626 (69.2%)	267 (29.5%)	101 (11.1%)	258 (28.5%)
Concurrent chemotherapy					
	No	388 (42.9%)	173 (19.1%)	68 (7.5%)	147 (16.2%)
	Yes	517 (57.1%)	238 (26.3%)	77 (8.5%)	202 (22.3%)
RT dose (Gy)					
	< 59.75	168 (18.6%)	95 (10.5%)	21 (2.3%)	52 (5.7%)
	≥ 59.75	737 (81.4%)	316 (34.9%)	124 (13.7%)	297 (32.8%)
Chemotherapy cycles					
	< 4	703 (77.7%)	312 (34.5%)	111 (12.3%)	280 (39.2%)
	≥ 4	202 (22.3%)	99 (10.9%)	34 (3.8%)	69 (7.6%)
Tumor location					
	Cervical/Upper	339 (37.5%)	134 (14.8%)	51 (5.6%)	154 (17.0%)
	Middle/Lower	566 (62.5%)	277 (30.6%)	94 (10.4%)	195 (21.5%)
Tumor length (cm)					
	< 5.85	506 (55.9%)	191 (21.1%)	88 (9.7%)	227 (25.2%)
	≥ 5.85	399 (44.1%)	220 (24.3%)	57 (6.3%)	122 (13.5%)
Tumor thickness (cm)					
	< 1.25	338 (37.3%)	85 (9.4%)	64 (7.1%)	189 (20.9%)
	≥ 1.25	567 (62.7%)	326 (36.0%)	81 (9.0%)	160 (17.8%)
T stage					
	T2/3	489 (54.0%)	195 (21.5%)	78 (8.6%)	216 (23.9%)
	T4	416 (46.0%)	216 (23.9%)	67 (7.4%)	133 (14.7%)
N stage					
	N0/1	634 (70.1%)	247 (27.3%)	95 (10.5%)	292 (32.3%)
	N2/3	271 (29.9%)	164 (18.1%)	50 (5.5%)	57 (6.3%)
TNM stage					
	Stage II/III	444 (49.1%)	166 (18.3%)	68 (7.5%)	210 (23.2%)
	Stage IVA	461 (50.9%)	245 (27.1%)	77 (8.5%)	139 (15.4%)
BMI					
	< 20.22	322 (35.6%)	179 (19.8%)	59 (6.5%)	84 (9.3%)
	≥ 20.22	583 (64.4%)	232 (25.6%)	86 (9.5%)	265 (29.3%)
SII					
	< 572.82	471 (52.0%)	160 (17.7%)	82 (9.0%)	229 (25.3%)
	≥ 572.82	434 (48.0%)	251 (27.7%)	63 (7.0%)	120 (13.3%)
SIRI					
	< 1.16	520 (57.5%)	188 (20.8%)	81 (9.0%)	251 (27.7%)
	≥ 1.16	385 (42.5%)	223 (24.6%)	64 (7.1%)	98 (10.8%)
NLR					
	< 2.20	405 (44.8%)	141 (15.6%)	69 (7.6%)	195 (21.5%)
	≥ 2.20	500 (55.2%)	270 (29.8%)	76 (8.4%)	154 (17.0%)
dNLR					
	< 0.83	238 (26.3%)	94 (10.4%)	46 (5.1%)	98 (10.8%)
	≥ 0.83	667 (73.7%)	317 (35.0%)	99 (10.9%)	251 (27.7%)
LMR					
	< 3.74	399 (44.1%)	221 (24.4%)	62 (6.9%)	116 (12.8%)
	≥ 3.74	506 (55.9%)	190 (21.0%)	83 (9.2%)	233 (25.7%)
NMLR					
	< 0.61	540 (59.7%)	281 (31.0%)	84 (9.3%)	175 (19.3%)
	≥ 0.61	365 (40.3%)	130 (14.4%)	61 (6.7%)	174 (19.2%)
PLR					
	< 142.52	507 (56.0%)	196 (21.7%)	89 (9.8%)	222 (24.5%)
	≥ 142.52	398 (44.0%)	215 (23.8%)	56 (6.2%)	127 (14.0%)
PNI					
	< 46.45	365 (40.3%)	191 (21.1%)	61 (6.7%)	113 (12.5%)
	≥ 46.45	540 (59.7%)	220 (24.3%)	84 (9.3%)	236 (26.1%)
ALI					
	< 347.97	438 (48.4%)	247 (27.3%)	68 (7.5%)	123 (13.6%)
	≥ 347.97	467 (51.6%)	164 (18.1%)	77 (8.5%)	226 (25.0%)
PAR					
	< 5.35	295 (32.6%)	94 (10.4%)	41 (4.5%)	160 (17.7%)
	≥ 5.35	610 (67.4%)	317 (35.0%)	104 (11.5%)	189 (20.9%)
PIV					
	< 247.55	459 (50.7%)	156 (17.2%)	68 (7.5%)	235 (26.0%)
	≥ 247.55	446 (49.3%)	255 (28.2%)	77 (8.5%)	114 (12.6%)
GNRI					
	< 96.56	434 (48.0%)	238 (26.3%)	79 (8.7%)	117 (12.9%)
	≥ 96.56	471 (52.0%)	173 (19.1%)	66 (7.3%)	232 (25.6%)

BMI, body mass index; SII, systemic immune-inflammation index; SIRI, systemic inflammatory response index; NLR, neutrophil-to-lymphocyte ratio; dNLR, derived neutrophil-to-lymphocyte ratio; LMR, lymphocyte-to-monocyte ratio; NMLR, neutrophil to monocyte to lymphocyte ratio; PLR, platelet-to-lymphocyte ratio; PNI, prognostic Nutritional Index; ALI, advanced lung cancer inflammation index; PAR, platelet-to-Albumin Ratio; PIV, pan-Immune-Inflammation Value; GNRI, geriatric nutritional risk index.

### Identification of prognostic risk factors based on consensus variables and multivariate Cox analysis and interpretation by SHAP model

Employing six distinct machine learning algorithms—LASSO, ElasticNet, SVM_Weight, GBM, Random Forest, and XGBoost—we conducted an initial screening of candidate variables to identify robust and reproducible predictors (**Figure 2A**). This process yielded a consensus set of 12 variables, each selected by at least three of the methods, which included sex, RT dose, N stage, tumor length, tumor thickness, platelet-to-Albumin Ratio (PAR), pan-Immune-Inflammation Value (PIV), systemic Immune-Inflammation Index (SII), platelet-to-Lymphocyte Ratio (PLR), prognostic Nutritional Index (PNI), geriatric nutritional risk index (GNRI), and advanced lung cancer inflammation index (ALI), (**Figure 2B**). This consensus approach ensured the robustness of variables carried forward for subsequent validation. Subsequently, these 12 consensus variables were entered into a multivariate Cox proportional hazards analysis to ascertain independent prognostic risk factors. The resulting forest plot identified three variables significantly associated with patient prognosis (P < 0.05): RT dose [hazard ratio (HR) = 0.710, P <0.05], tumor thickness (HR = 2.463, P < 0.001), and N stage (HR = 1.754, P < 0.001) (**Figure 2C**).

**Figure 2:**
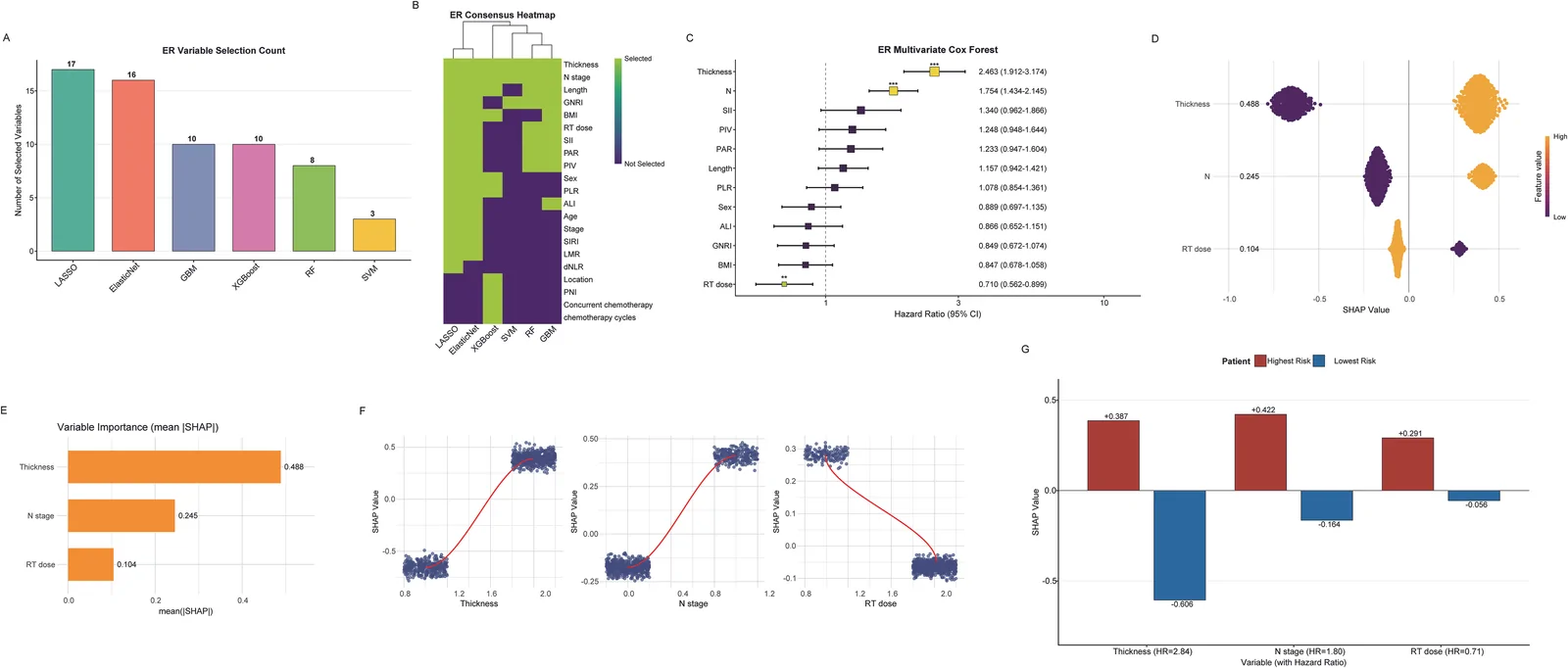
Identification and interpretation of independent risk factors for ER. **(A, B)** Consensus-based feature selection across six machine learning algorithms. **(C)** Multivariable Cox regression identifying three independent ER risk factors: RT dose, tumor thickness, and N stage. **(D–G)** SHAP analysis validating and interpreting the influence of the identified factors on individual risk predictions.

To elucidate the contribution of these risk factors within the predictive model, we performed a model interpretability analysis using SHAP values. The SHAP summary plot, ranking features by their mean absolute SHAP value, confirmed that the three most influential variables on the model output were tumor thickness (mean |SHAP| = 0.488), N stage (0.240), and RT dose (0.104), aligning with their significance in the Cox model (**Figures 2D, E**). SHAP dependence plots confirmed the directional effects of the three predictors: higher tumor thickness and higher N stage increased the predicted risk of early recurrence (positive SHAP values), whereas higher radiotherapy dose decreased the risk (negative SHAP values), consistent with their hazard ratios (HR 2.46, 1.75, and 0.71, respectively) (**Figure 2F**). Finally, by contrasting SHAP force waterfall plots for patients with the highest and lowest predicted risk, we visualized how these key variables drive individualized risk predictions, thereby providing patient-level explanations for the model’s output (**Figure 2G**).

Similarly, we first identified a consensus variable set comprising 9 factors: PAR, BMI, concurrent chemotherapy, N stage, TNM stage, sex, tumor thickness, PIV, and GNRI (**Figures 3A, B**). Subsequent multivariable Cox analysis established four independent risk factors for LR: N stage (HR = 2.759, P<0.001), PIV (HR = 1.517, P<0.05), PAR (HR = 1.502, P<0.05), and concurrent chemotherapy (HR = 0.699, P<0.05) (**Figure 3C**). SHAP interpretability analysis further confirmed that these four factors exerted the strongest positive driving effects on the predictive model, with mean absolute SHAP values of 0.437, 0.274, 0.233, and 0.191 for N stage, PIV, PAR, and concurrent chemotherapy, respectively (**Figures 3D–G**).

**Figure 3:**
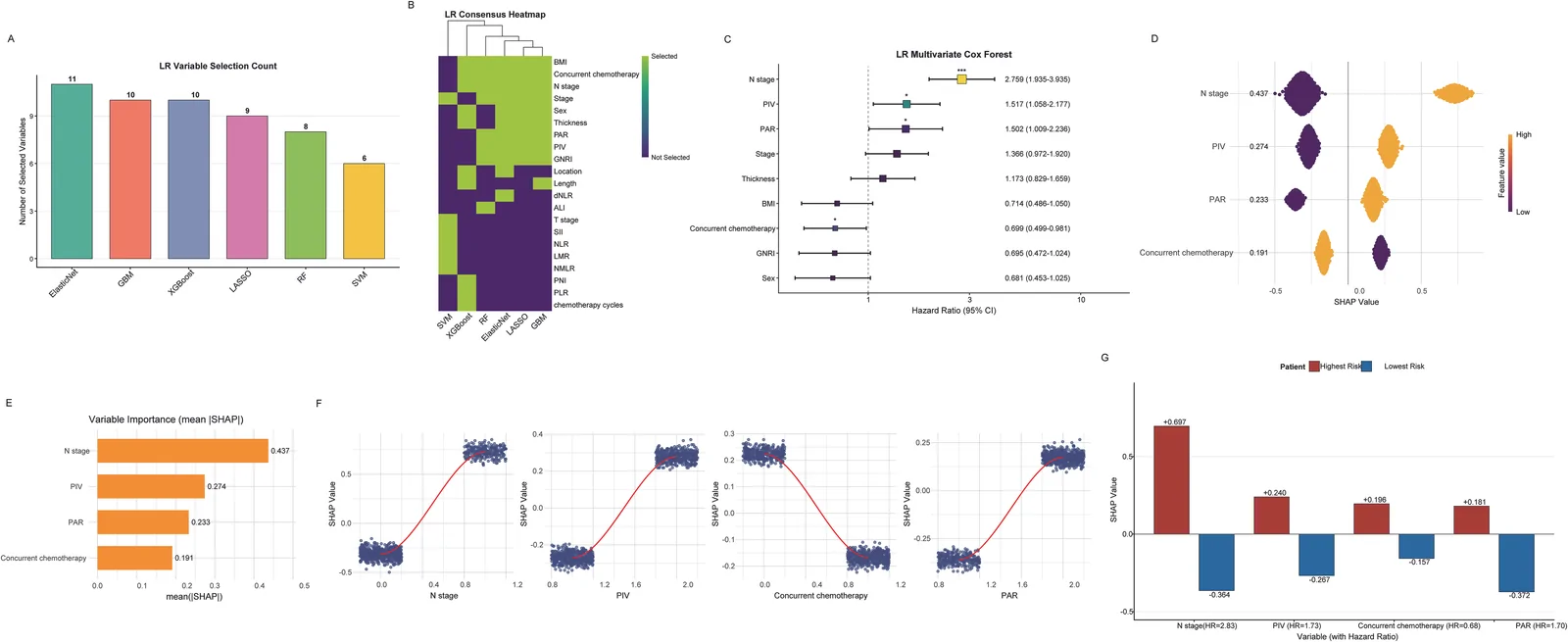
Identification and interpretation of independent risk factors for LR. **(A, B)** Consensus-based feature selection across six machine learning algorithms. **(C)** Multivariable Cox regression identifying four independent LR risk factors: PAR, PIV, N stage, and concurrent chemotherapy. **(D–G)** SHAP analysis validating and interpreting the influence of the identified factors on individual risk predictions.

### Development and validation of a prognostic nomogram

We integrated the independent risk factors for both ER and LR to construct a nomogram model for predicting prognosis in the entire study cohort. The model demonstrated favorable performance in forecasting both OS and PFS (**Figure 4A**). The 1-, 3-, and 5-year calibration curves for both OS and PFS showed high concordance between the predicted probabilities and the actually observed outcomes (**Figure 4B**). Decision curve analysis at the corresponding time points indicated that the model provided a higher net clinical benefit than any single independent variable (**Figure 4C**). Regarding discriminatory ability, the model also displayed superior predictive accuracy: the time dependent AUC values for OS at 1, 3, and 5 years were 0.709, 0.709, and 0.738, respectively, while the corresponding AUC values for PFS were 0.712, 0.738, and 0.771. All AUC values were higher than those of the individual risk factors (**Figure 4D**). The C-index of model was 0.652 and 0.677 for OS and PFS.

**Figure 4:**
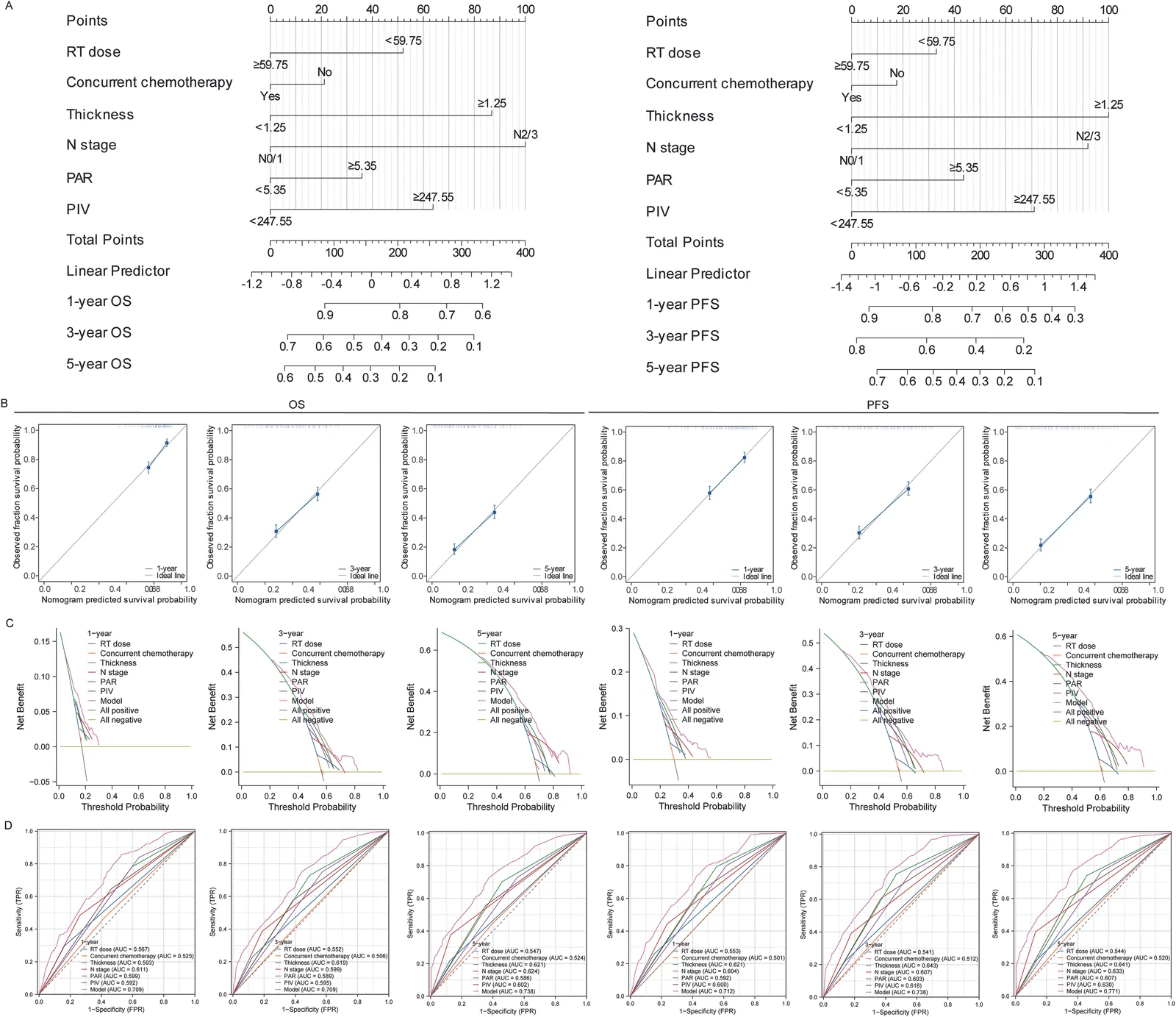
Development and validation of the prognostic nomogram. **(A)** Nomogram integrating the independent risk factors for predicting OS and PFS. **(B)** Calibration curves for OS and PFS at 1, 3, and 5 years. **(C)** Decision curve analysis evaluating the net clinical benefit of the nomogram versus individual variables for OS and PFS. **(D)** Time-dependent ROC curves at 1, 3, and 5 years for OS and PFS, demonstrating superior discriminatory ability of the nomogram compared to single factors.

### Clinical characteristics of ER, LR, and different risk groups

Kaplan–Meier analysis demonstrated that patients with ER had significantly worse OS and PFS than those with LR (**Figures 5A, B**), and the accompanying heatmap showed that the early-recurrence group had more advanced tumor stage and greater tumor burden, consistent with its poorer prognosis (**Figure 5C**). Subsequently, the entire cohort was divided into low-, moderate-, and high-risk groups according to tertiles of the nomogram score (range, 0–361). Kaplan–Meier curves revealed that the high-risk group had significantly worse survival than the low-risk group, a gradient that was consistently reflected in the enrichment of adverse clinicopathological features in the corresponding heatmap (**Figures 5D–F**).

**Figure 5:**
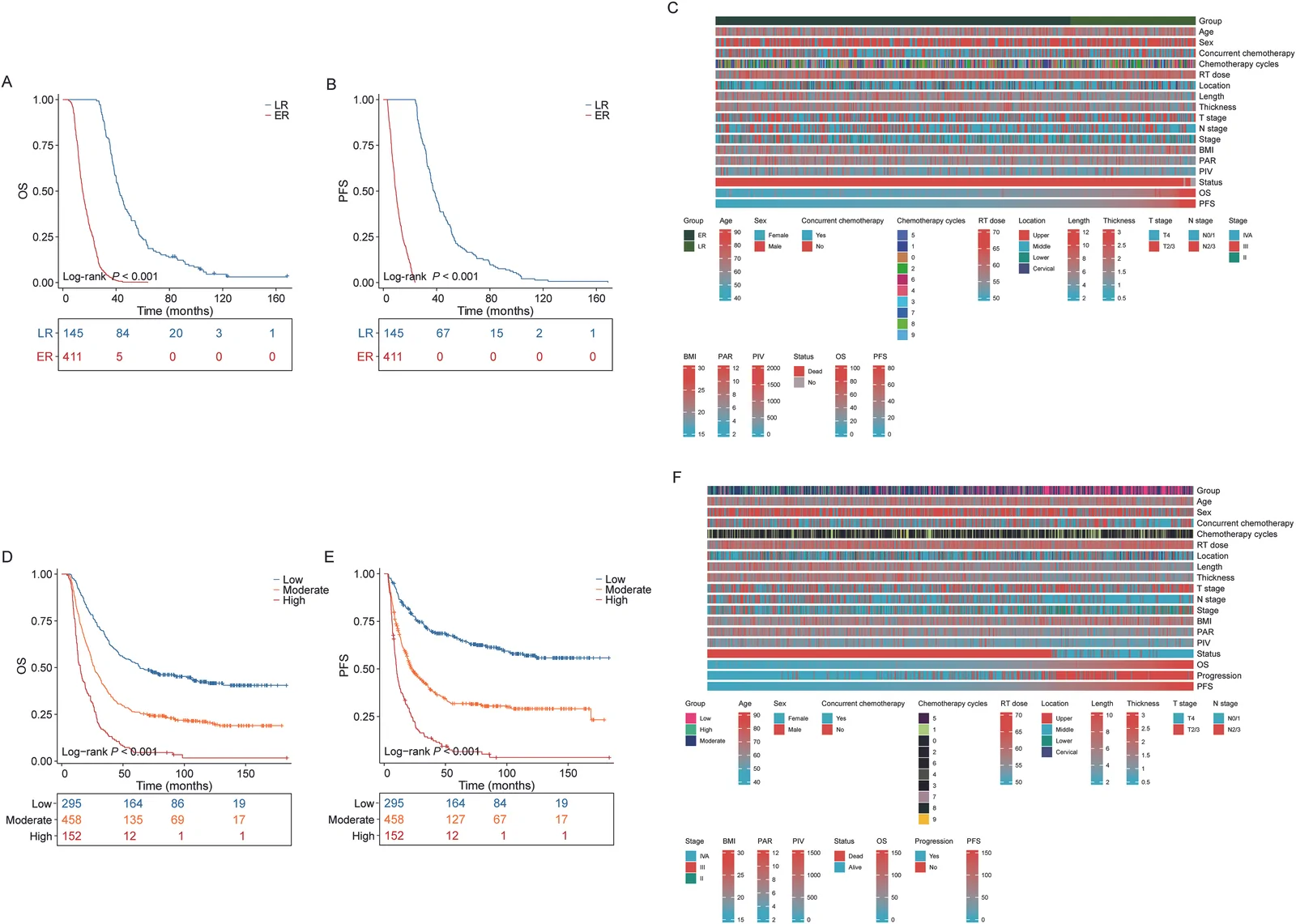
**(A, B)** Comparison of OS and PFS between ER and LR. **(C)** Heatmap of the distribution of key clinicopathological variables in ER and LR. **(D, E)** Comparison of OS and PFS among different risk groups. **(F)** Heatmap of the distribution of key clinicopathological variables among different risk groups.

### Risk-stratified follow-up strategy informed by restricted mean survival time analysis

The full model, which integrated the ER and LR risk factors, significantly improved the prediction of 5-year outcomes over either model alone. Compared with the ER model, the full model increased the category-free NRI by 0.400 and the IDI by 0.034 for OS, and by 0.514 and 0.055 for PFS; compared with the LR model, the corresponding improvements were 0.472 and 0.040 for OS, and 0.569 and 0.053 for PFS (**Figure 6A**).

**Figure 6:**
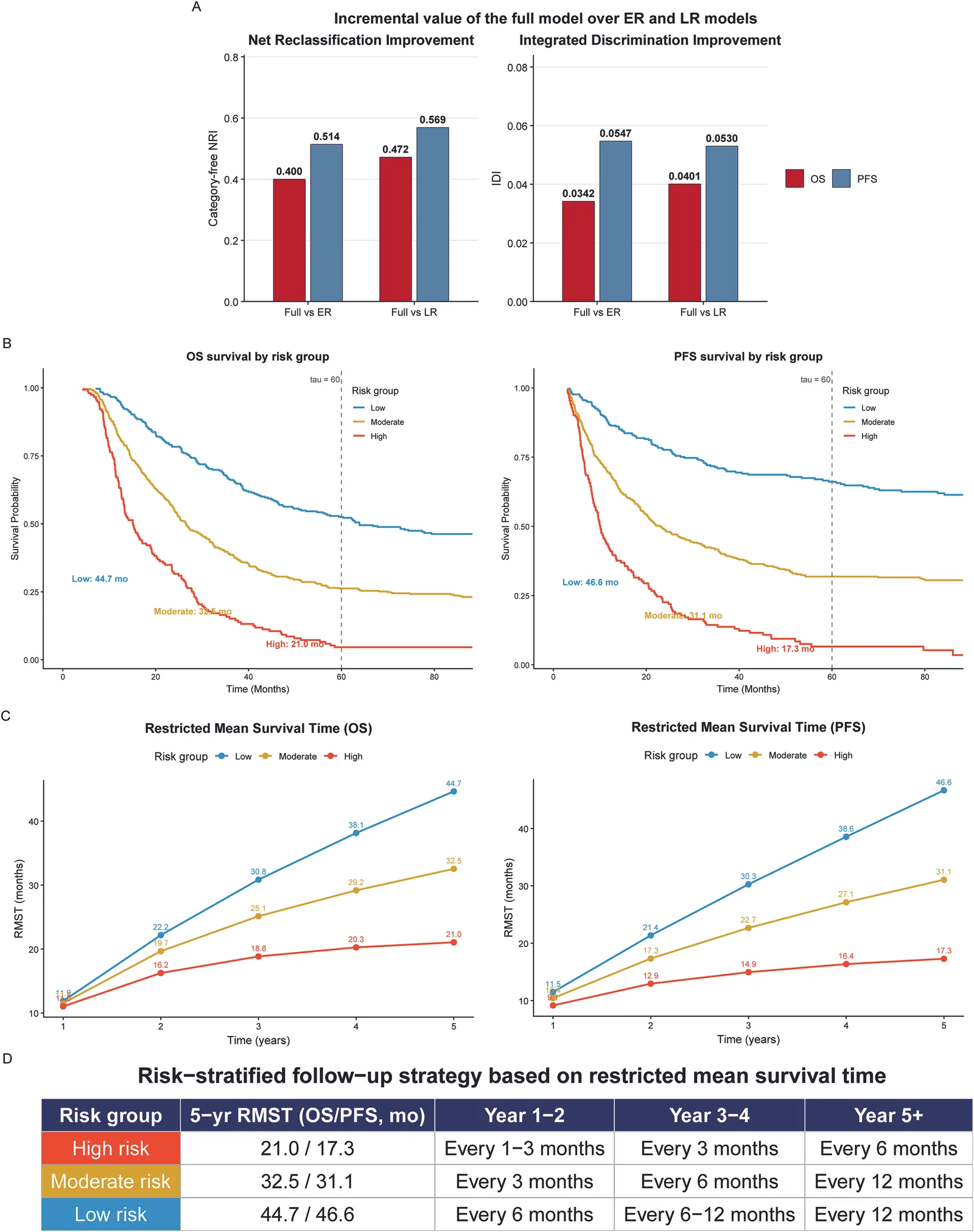
The survival differences were quantified using RMST and the derived risk-adaptive follow-up strategy. **(A)** NRI and IDI analyses of the full model compared to ER and LR. **(B)** The RMST values of OS and PFS in the low, moderate, and high-risk subgroups at 5 years. **(C)** It shows the RMST values of different risk groups over 1–5 years. **(D)** Based on this quantified difference, a risk stratification follow-up framework was proposed.

Based on the analysis of the RMST, we identified a significant gradient in survival time among low-, moderate-, and high-risk patients. For instance, at the 5-year observation time point, the RMST of the high-risk group was significantly shorter than that of the moderate- and low-risk groups (OS-RMST: approximately 21.0 months for high-risk, 32.5 months for moderate-risk, and 44.7 months for low-risk; PFS-RMST: approximately 17.3 months for high-risk, 31.1 months for moderate-risk, and 46.6 months for low-risk) (**Figures 6B, C**). These findings indicate that high-risk patients not only have a higher risk of recurrence but also a markedly shorter average survival time. Informed by this survival disparity, we developed a risk-stratified, individualized follow-up strategy. For the high-risk group, due to early and concentrated event occurrence, an intensive follow-up schedule is recommended, with comprehensive reassessment every 1–3 months. The moderate-risk group is advised to adopt a step-down follow-up approach: every 3 months during the first 1–2 years after treatment, adjusted to every 6 months during years 2–4, and then extended to every 12 months or annually from the fifth year onward. For the low-risk group, follow-up should be conducted every six months during the first two years and then annually thereafter, with a focus on long-term quality of life (**Figure 6D**).

## Discussion

This study conducted a comprehensive analysis of the heterogeneity in disease progression among patients with locally advanced ESCC who underwent dCRT or dRT. By employing a novel two-stage analytical strategy, we first integrated a consensus-based machine learning approach for feature selection, ensuring robustness in the initial variable screening and mitigating potential bias from any single method. Subsequently, Cox regression analysis was performed to further identify key risk factors. The results revealed that N stage, RT dose, and tumor thickness were the main drivers of ER, whereas N stage, PIV, PAR, and concurrent chemotherapy were the core factors for LR. To enhance model interpretability, SHAP analysis was introduced to visualize the direction and relative importance of each risk factor at both cohort and individual patient levels. Based on these findings, we developed an integrative nomogram as a crucial tool for clinical translation. This nomogram demonstrated moderate performance in calibration and discrimination and showed higher clinical net benefit compared to any single variable, confirming its practical utility in predicting PFS and OS. The survival curves were clearly separated between risk groups defined by the nomogram. Finally, follow-up strategies for different risk stratifications are formulated based on the RMST analysis. The findings of this study address a critical gap in the current management paradigm for ESCC and provide a solid framework for individualized patient monitoring and potential therapeutic interventions.

Our analysis confirms that lymph node stage and PAR are cornerstone factors influencing the prognosis of ESCC patients, with their impact persisting throughout the course of disease recurrence. The N stage directly quantifies the extent of lymphatic metastasis, and both this study and previous data consistently demonstrate that the presence and extent of lymph node metastasis are significantly negatively correlated with survival rates, reinforcing its central role in any risk model (21, 22). On the other hand, PAR, as a composite indicator capturing both systemic inflammatory status and nutritional condition, highlights the critical role of the “inflammation-nutrition” axis in the tumor microenvironment. Chronic inflammation promotes tumor progression by driving proliferation, angiogenesis, and immune suppression, while malnutrition weakens the patient’s tolerance and recovery capacity for aggressive treatments (23–26). Thus, elevated PAR signals an unfavorable state characterized by both pro-tumor activity and systemic vulnerability, which is closely associated with poorer survival outcomes.

Notably, this study further elucidates the specific effects of different factors on the temporal patterns of recurrence. Tumor thickness is primarily associated with early recurrence, as thicker tumors often possess stronger local invasive capacity, readily infiltrate vascular structures, provide physical channels for early dissemination of tumor cells, and reflect their inherent high proliferative and metastatic potential (27–29). In contrast, the PIV shows a significant correlation with late recurrence. The sustained systemic inflammatory response represented by PIV may suppress immune surveillance and maintain a tumor-promoting microenvironment, thereby “awakening” dormant micro-metastases and leading to long-term treatment failure (30–32). Clarifying this temporal heterogeneity suggests that intervention strategies for early and late recurrence may need to be tailored differently.

The primary contribution of this study lies in translating the aforementioned exploration of biological mechanisms into a quantifiable tool with potential for clinical translation. The nomogram model we developed is not merely a simple additive aggregation of risk factors. Instead, it organically integrates these biologically complementary metrics by combining machine learning with multivariate statistical methods. In internal validation, the model demonstrated the following characteristics: the calibration curve indicated good agreement between predicted and actual risk; the time-dependent AUC value reached 0.771, demonstrating the model’s relatively strong discriminative ability; and decision curve analysis suggested that, within a certain threshold probability range, using this model for clinical decision-making might yield a higher net benefit than relying on single indicators or empirical judgment. Furthermore, survival curves showed significant separation between different subgroups stratified according to the model’s risk score. Collectively, these results indicate that our research has evolved from identifying risk factors to achieving the interim goals of quantifying recurrence risk and stratifying patient risk levels.

Subsequently, based on different subgroups divided by the risk model, the RMST of each group was further quantified, and a set of risk-stratified and adaptive individualized follow-up strategies were formulated accordingly. Compared with the relatively unified follow-up protocols recommended by current conventional guidelines, this strategy shows significant differences both in concept and practice (4, 5, 33). Its core advantage lies in the transformation from “homogenization” to “precision”. For high-risk groups, more intensive monitoring (e.g., every 3 months) is recommended to detect early signs of recurrence; for low-risk groups, the interval between re-examinations is appropriately extended (e.g., every 6–12 months), which reduces unnecessary examination burdens and medical costs while ensuring safety. Meanwhile, the key follow-up time points in the strategy are directly derived from cohort data, reflecting the real disease progression kinetics of different risk populations. It is more evidence-driven than fixed intervals based on expert consensus and helps optimize the allocation efficiency of medical resources. However, the clinical application of this strategy also faces several challenges. Its effectiveness highly depends on the accuracy and robustness of the risk-stratification model, and external validation in a broader population and more institutions is necessary to ensure its universality. In addition, compared with the simple and clear unified guidelines, the implementation of the stratified strategy is more complex, requiring doctors to perform additional score calculations, grouping, and in-depth communication with patients. The optimal follow-up intensity for patients in the intermediate-risk group still needs further clarification and validation. Despite these limitations, this strategy represents a tangible step towards precision medicine in esophageal cancer management. It provides, for the first time, a quantitative and operable framework for the differential follow-up of patients with locally advanced esophageal squamous cell carcinoma who have received dCRT, directly translating the prognostic research results into clinical action plans. In the future, by integrating it into the electronic medical record system and verifying whether it can improve the early diagnosis rate of high-risk patients or the quality of life of low-risk patients in prospective applications, this strategy is expected to provide a promising path for promoting guideline updates and achieving truly individualized patient management.

Despite our efforts, it must be acknowledged that this study has certain limitations. First, as a single-center retrospective study, it is inevitably affected by selection bias and unmeasured confounding factors, even though we employed rigorous statistical methods such as IPTW to mitigate confounding. Second, the analysis relied on routine clinicopathological variables, and potentially important information such as genomic features and detailed treatment parameters was not included. Third, inflammatory biomarkers are influenced by multiple non-cancer conditions, including chronic inflammation, nutritional status, infection, and other systemic illnesses. Fourth, Furthermore, the long inclusion period of this study encompasses changes in radiotherapy techniques, staging criteria, and supportive care. These unmeasured time-related factors may have influenced the study results. Finally, although the model performed well in internal validation, its universality and stability in independent, multi-center cohorts still need to be confirmed through external validation.

## Conclusion

This study systematically revealed the heterogeneity of disease recurrence in locally advanced ESCC after dCRT, identifying RT dose, concurrent chemotherapy, N stage, PAR, tumor thickness, and PIV as key recurrence predictors. By integrating machine learning and multivariate analysis, we constructed and internally validated a prognostic nomogram that accurately stratifies individual recurrence risks. Based on RMST-derived survival differences, we propose a risk-adapted follow-up framework that provides a basis for future prospective evaluation of individualized surveillance strategies. This framework lays the groundwork for optimizing post-treatment monitoring and developing risk-stratified treatment approaches, pending external validation.

## Data Availability

The data supported in this study are available upon reasonable request by the corresponding author.

## References

[1] Siegel RL Giaquinto AN Jemal A . Cancer statistics, 2024. CA Cancer J Clin. (2024) 74:12–49. doi: 10.3322/caac.21820 38230766

[2] Xia C Dong X Li H Cao M Sun D He S . Cancer statistics in China and United States, 2022: profiles, trends, and determinants. Chin Med J (Engl). (2022) 135:584–90. doi: 10.1097/cm9.0000000000002108 35143424 PMC8920425

[3] Abnet CC Arnold M Wei WQ . Epidemiology of esophageal squamous cell carcinoma. Gastroenterology. (2018) 154:360–73. doi: 10.1053/j.gastro.2017.08.023 28823862 PMC5836473

[4] Ajani JA D'Amico TA Bentrem DJ Cooke D Corvera C Das P . Esophageal and esophagogastric junction cancers, version 2.2023, NCCN clinical practice guidelines in oncology. J Natl Compr Canc Netw. (2023) 21:393–422. doi: 10.6004/jnccn.2023.0019 37015332

[5] Obermannova R Alsina M Cervantes A Leong T Lordick F Nilsson M . Oesophageal cancer: ESMO clinical practice guideline for diagnosis, treatment and follow-up. Ann Oncol. (2022) 33:992–1004. doi: 10.1016/j.annonc.2022.07.003 35914638

[6] Zhou H Tan L Shen Y Jun Y Liu T Ai L . Long-survival of a patient with esophageal cancer benefited from comprehensive treatment and MDT: a case report. J Thorac Dis. (2024) 16:8110–6. doi: 10.21037/jtd-24-227 39678875 PMC11635259

[7] Kaida H Kitajima K Nakajo M Ishibashi M Matsunaga T Minamimoto R . Predicting tumor response and prognosis to neoadjuvant chemotherapy in esophageal squamous cell carcinoma patients using PERCIST: a multicenter study in Japan. Eur J Nucl Med Mol Imaging. (2021) 48:3666–82. doi: 10.1007/s00259-021-05365-5 33934168

[8] Zhao YX Zhao HP Zhao MY Yu Y Qi X Wang JH . Latest insights into the global epidemiological features, screening, early diagnosis and prognosis prediction of esophageal squamous cell carcinoma. World J Gastroenterol. (2024) 30:2638–56. doi: 10.3748/wjg.v30.i20.2638 38855150 PMC11154680

[9] Gao Y Li LX Rowland A Karapetis CS Parent N Kichenadasse G . Progressive lesion type is predictive of post-progression survival in first-line chemoimmunotherapy for Non-Small Cell Lung Cancer. Cancer Lett. (2026) 639:218191. doi: 10.1016/j.canlet.2025.218191 41386504

[10] Yang JJ Chen HJ Yan HH Zhang XC Zhou Q Su J . Clinical modes of EGFR tyrosine kinase inhibitor failure and subsequent management in advanced non-small cell lung cancer. Lung Cancer. (2013) 79:33–9. doi: 10.1016/j.lungcan.2012.09.016 23079155

[11] Isaka T Ito H Nakayama H Yokose T Saito H Masuda M . Impact of the initial site of recurrence on prognosis after curative surgery for primary lung cancer. Eur J Cardiothorac Surg. (2022) 61:778–86. doi: 10.1093/ejcts/ezab442 34686875

[12] Parry K Visser E van Rossum PS Mohammad NH Ruurda JP van Hillegersberg R . Prognosis and treatment after diagnosis of recurrent esophageal carcinoma following esophagectomy with curative intent. Ann Surg Oncol. (2015) 22:S1292–300. doi: 10.1245/s10434-015-4840-5 26334295 PMC4686569

[13] Kim S Shin J Kim DY Choi GH Kim MJ Choi JY . Radiomics on gadoxetic acid-enhanced magnetic resonance imaging for prediction of postoperative early and late recurrence of single hepatocellular carcinoma. Clin Cancer Res. (2019) 25:3847–55. doi: 10.1158/1078-0432.ccr-18-2861 30808773

[14] Takeshita T Yan L Asaoka M Rashid O Takabe K . Late recurrence of breast cancer is associated with pro-cancerous immune microenvironment in the primary tumor. Sci Rep. (2019) 9:16942. doi: 10.1038/s41598-019-53482-x 31729458 PMC6858361

[15] Chen MC Su HY Su YH Huang KH Fang WL Lin CW . The clinicopathological and genetic differences among gastric cancer patients with no recurrence, early recurrence, and late recurrence after curative surgery. J Chin Med Assoc. (2023) 86:57–64. doi: 10.1097/jcma.0000000000000846 36374529 PMC12755660

[16] Sheridan RP Wang M Liaw A Ma J Gifford E . Correction to extreme gradient boosting as a method for quantitative structure-activity relationships. J Chem Inf Model. (2020) 60:1910. doi: 10.1021/acs.jcim.0c00029 31975583

[17] Zeleke AJ Palumbo P Tubertini P Miglio R Chiari L . Machine learning-based prediction of hospital prolonged length of stay admission at emergency department: a gradient boosting algorithm analysis. Front Artif Intell. (2023) 6:1179226. doi: 10.3389/frai.2023.1179226 37588696 PMC10426288

[18] Hong X Chen S Harris CJ . Elastic-net prefiltering for two-class classification. IEEE Trans Cybern. (2013) 43:286–95. doi: 10.1109/tsmcb.2012.2205677 22829416

[19] Qi B Zhao C Yin G . Feature weighting algorithms for classification of hyperspectral images using a support vector machine. Appl Opt. (2014) 53:2839–46. doi: 10.1364/ao.53.002839 24921869

[20] Han K Jung I . Restricted mean survival time for survival analysis: a quick guide for clinical researchers. Korean J Radiol. (2022) 23:495–9. doi: 10.3348/kjr.2022.0061 35506526 PMC9081686

[21] Dedavid e Silva TL Damin DC . Lymph node ratio predicts tumor recurrence in stage III colon cancer. Rev Col Bras Cir. (2013) 40:463–70. doi: 10.1590/s0100-69912013000600008 24573624

[22] Shen X Chen T Yu J Wen J Ji H Guo Z . Survival effect of PET/CT-defined occult lymph node metastasis in the newly proposed ninth edition N descriptors: a multicentre study. Eur J Nucl Med Mol Imaging. (2026) 53:1729–38. doi: 10.1007/s00259-025-07544-0 40986090

[23] Grivennikov SI Greten FR Karin M . Immunity, inflammation, and cancer. Cell. (2010) 140:883–99. doi: 10.1016/j.cell.2010.01.025 20303878 PMC2866629

[24] Diakos CI Charles KA McMillan DC Clarke SJ . Cancer-related inflammation and treatment effectiveness. Lancet Oncol. (2014) 15:e493–503. doi: 10.1016/s1470-2045(14)70263-3 25281468

[25] Stumpf F Keller B Gressies C Schuetz P . Inflammation and nutrition: Friend or foe? Nutrients. (2023) 15(5):1159. doi: 10.3390/nu15051159 36904164 PMC10005147

[26] Nicolini A Ferrari P Masoni MC Fini M Pagani S Giampietro O . Malnutrition, anorexia and cachexia in cancer patients: a mini-review on pathogenesis and treatment. BioMed Pharmacother. (2013) 67:807–17. doi: 10.1016/j.biopha.2013.08.005 24035652

[27] Pinto FR de Matos LL Palermo FC Kulcsar MA Cavalheiro BG de Mello ES . Tumor thickness as an independent risk factor of early recurrence in oral cavity squamous cell carcinoma. Eur Arch Otorhinolaryngol. (2014) 271:1747–54. doi: 10.1007/s00405-013-2704-9 24071858

[28] Son HJ Lee YS Ku JY Roh JL Choi SH Nam SY . Radiological tumor thickness as a risk factor for local recurrence in early glottic cancer treated with laser cordectomy. Eur Arch Otorhinolaryngol. (2018) 275:153–60. doi: 10.1007/s00405-017-4793-3 29098372

[29] Ganly I Goldstein D Carlson DL Patel SG O'Sullivan B Lee N . Long-term regional control and survival in patients with "low-risk," early stage oral tongue cancer managed by partial glossectomy and neck dissection without postoperative radiation: the importance of tumor thickness. Cancer. (2013) 119:1168–76. doi: 10.1002/cncr.27872 23184439

[30] Cheng X Luo K Gao Y Wang R Liu S Li Q . Pan-immune-inflammation value predicts survival of patients with oesophageal squamous cell carcinoma receiving immunotherapy and chemoradiotherapy: a pooled analysis of two phase II trials. Ann Med. (2025) 57:2576639. doi: 10.1080/07853890.2025.2576639 41123456 PMC12548070

[31] Feng J Wang L Yang X Chen Q Cheng X . Pretreatment pan-immune-inflammation value (PIV) in predicting therapeutic response and clinical outcomes of neoadjuvant immunochemotherapy for esophageal squamous cell carcinoma. Ann Surg Oncol. (2024) 31:272–83. doi: 10.1245/s10434-023-14430-2 37838648

[32] Nishida A Andoh A . The role of inflammation in cancer: mechanisms of tumor initiation, progression, and metastasis. Cells. (2025) 14(7):488. doi: 10.3390/cells14070488 40214442 PMC11987742

[33] Stahl M Oliveira J Group EGW . Esophageal cancer: ESMO clinical recommendations for diagnosis, treatment and follow-up. Ann Oncol. (2008) 19:ii21–2. doi: 10.1093/annonc/mdm018 18456754

