# Supplementary material for: Development of a risk-stratified follow-up strategy based on early and late recurrence risk factors in locally advanced esophageal squamous cell carcinoma after definitive chemoradiotherapy
Source: Front Oncol. 2026 Sep 23;16:1903732. doi: 10.3389/fonc.2026.1903732 (PMC13642816; doi:10.3389/fonc.2026.1903732)
Supplement: Supplementary file 1 [file Table1.docx]

**Formulas for Inflammatory and Nutritional Indices**

**Inflammatory Indices**

Systemic Immune-Inflammation Index (SII): SII = (Neutrophil count × Platelet count ) / Lymphocyte count

Systemic Inflammatory Response Index (SIRI): SIRI = (Neutrophil count × Monocyte count ) / Lymphocyte count

Neutrophil-to-Lymphocyte Ratio (NLR): NLR = Neutrophil count / Lymphocyte count

Derived Neutrophil-to-Lymphocyte Ratio (dNLR): dNLR = Neutrophil count / (White blood cell count – Lymphocyte count )

Lymphocyte-to-Monocyte Ratio (LMR): LMR = Lymphocyte count / Monocyte count

Neutrophil to Monocyte to Lymphocyte Ratio (NMLR): NMLR = (Monocyte count + Lymphocyte count ) / Neutrophil count

Platelet-to-Lymphocyte Ratio (PLR): PLR = Platelet count / Lymphocyte count Pan-Immune-Inflammation Value (PIV): PIV = (Neutrophil count × Platelet count × Monocyte count × 10⁻¹⁸) / Lymphocyte count

**Nutritional Indices**

Prognostic Nutritional Index (PNI): PNI = Albumin + 5 × Lymphocyte count

Body Mass Index (BMI): BMI = Weight / Height (m)²

Advanced Lung cancer inflammation Index (ALI): ALI = BMI × Albumin / NLR

Platelet-to-Albumin Ratio (PAR): PAR = Platelet count / Albumin

Geriatric Nutritional Risk Index (GNRI): GNRI = 1.489 × Albumin + 41.7 × Weight / Ideal Weight ; Ideal Weight (IW) = 22 × Height (m)²

If the actual weight (W) is greater than the ideal weight (IW), then the ratio (W/IW) is set to 1 for the calculation.
